# A pathogenicity locus of *Streptococcus gallolyticus* subspecies *gallolyticus*

**DOI:** 10.1038/s41598-023-33178-z

**Published:** 2023-04-18

**Authors:** John Culver Taylor, Ritesh Kumar, Juan Xu, Yi Xu

**Affiliations:** 1 Center for Infectious and Inflammatory Diseases, Texas A&M Health Science Center Institute of Biosciences of Technology, Houston, TX USA; 2 Department of Microbiology and Molecular Genetics, McGovern Medical School, UT Health, Houston, TX USA; 3 Department of Microbial Pathogenesis and Immunology, College of Medicine, Texas A&M Health Science Center, Texas, USA; 4 Present Address: IFF Health and Biosciences, Madison, USA

**Keywords:** Microbiome, Microbiology, Bacteriology, Gastrointestinal cancer

## Abstract

*Streptococcus gallolyticus* subspecies *gallolyticus* (*Sgg*) is known to be strongly associated with colorectal cancer (CRC). Recent functional studies further demonstrated that *Sgg* actively stimulates CRC cell proliferation and promotes the development of colon tumors. However, the *Sgg* factors important for the pro-proliferative and pro-tumor activities of *Sgg* remain unclear. Here, we identified a chromosomal locus in *Sgg* strain TX20005. Deletion of this locus significantly reduced *Sgg* adherence to CRC cells and abrogated the ability of *Sgg* to stimulate CRC cell proliferation. Thus, we designate this locus as the *Sgg* pathogenicity-associated region (*SPAR*). More importantly, we found that *SPAR* is important for *Sgg* pathogenicity in vivo. In a gut colonization model, mice exposed to the *SPAR* deletion mutant showed significantly reduced *Sgg* load in the colonic tissues and fecal materials, suggesting that *SPAR* contributes to the colonization capacity of *Sgg*. In a mouse model of CRC, deletion of *SPAR* abolished the ability of *Sgg* to promote the development of colon tumors growth. Taken together, these results highlight *SPAR* as a critical pathogenicity determinant of *Sgg*.

## Introduction

*Streptococcus gallolyticus* subspecies *gallolyticus* (*Sgg*) is a group D streptococcal bacterium, and an opportunistic pathogen belonging to the *Streptococcus bovis/Streptococcus equinis* complex (SBSEC)^1^. *Sgg* is a causative agent of bacteremia and infective endocarditis (IE), and has a long-standing clinical association with CRC, such that numerous studies and case reports indicate that *Sgg* is associated with CRC, and certain *Sgg* strains are capable of stimulating cell proliferation and enhancing tumor burden^2–15^. Patients with *Sgg* IE and/or bacteremia have concomitant colon adenomas or adenocarcinomas at a much higher rate (~ 60%) than that of the general population^8,16^. Moreover, a prospective study found that 45% of patients with *Sgg* IE developed colonic neoplastic lesions within 5 years of IE diagnosis compared to 21% of patients with IE due to closely-related enterococci, further supporting the association of *Sgg* with CRC^6^. In CRC patients with no symptoms of IE or bacteremia, several studies of cohorts from different geographical locations showed that *Sgg* preferentially associates with tumor tissues compared to normal adjacent or normal tissues, or shows increased prevalence in stool samples from CRC patients^4,17–19^.

Functionally, *Sgg* stimulates the proliferation of human CRC cells in a cell context-dependent fashion^4,20^. In addition, studies have shown that there is phenotypic heterogeneity among *Sgg* strains with respect to the ability to stimulate host cell proliferation. Some strains of *Sgg*, such as TX20005, are able to stimulate cell proliferation, while others, such as ATCC_43143, are not^2,4,20^. Studies using the prototypic *Sgg* strain TX20005 also showed that stimulation of host cell proliferation by *Sgg* requires β-catenin in in vitro cultured cells and in a xenograft model in vivo. Exposure to *Sgg* also led to larger and more advanced tumors in an azoxymethane (AOM)-induced CRC model and in a colitis-associated CRC model^4,15^. Previous work also indicated that there are variations among *Sgg* strains in their ability to promote tumor growth. Some *Sgg* strains such as ATCC_43143 showed significantly reduced capacity to promote tumor development in vivo compared to TX20005^2^, suggesting that there are specific *Sgg* factors contributing to the pro-proliferative and pro-tumor activities of *Sgg*. The identity of these specific *Sgg* factors was unknown. Previous work suggested that effectors of a type VII secretion system (T7SS) of *Sgg* are important for these activities^3^.

Here, we describe work characterizing a chromosomal locus of *Sgg* strain TX20005. Deletion of this locus significantly impaired the ability of *Sgg* to adhere to cultured CRC cells and abrogated the ability of *Sgg* to stimulate CRC cell proliferation. In vivo, the deletion mutant displayed significantly reduced capacity to colonize normal mouse colons. More importantly, the mutant lost the ability to promote the development of colon tumors. Given the role of this locus in *Sgg* pathogenesis, we have coined this locus as the *Sgg* pathogenicity-associated region (*SPAR*).

## Materials and methods

### Bacterial strains, cell lines and growth conditions

*Sgg* strains were routinely grown in brain heart infusion (BHI) broth or tryptic soy broth at 37 °C with shaking, or on BHI or TSB agar plates at 37 °C overnight (Teknova). For co-culture experiments and animal studies, stationary phase bacterial cultures were pelleted, washed with phosphate-buffered saline (PBS), pH 7.4, resuspended in PBS containing 15% glycerol, aliquoted and stored at − 80 °C. Aliquoted stocks were thawed, washed with PBS, diluted in appropriate media to obtain the desired concentration and directly added to cells or administered to mice. Human colon cancer cell lines HT29, HCT116, and SW480, as well as the HEK293 cell line were grown in Dulbecco’s Modified Eagle’s Medium F-12 50/50 (DMEM/F-12, GIBCO) supplemented with 10% fetal bovine serum (FBS) at 37 °C with 5% CO_2_ in a humidified chamber. Cells from less than 30 passages were used in the experiments.

### Preparation of bacterial culture supernatants (CSs)

Supernatants from stationary phase *Sgg* cultured in BHI were filtered-sterilized through a 0.2 μm filter and concentrated approximately 10–20-fold using centrifugal concentrators (3kD molecular weight cut off (MWCO)). The concentrated CSs were aliquoted and stored at − 80 °C. A vial is thawed and diluted in the appropriate tissue culture media to 1X and then used immediately in adherence or cell proliferation assays. To determine the nature of the active components in the CSs, 10X concentrated CSs were treated with 50 mM trypsin, α-amylase, or lipase for 1 h at room temperature. CSs were then filtered through a 30kD MWCO centrifugal concentrator to separate the enzymes. The flow through was then concentrated again using a 3kD MWCO centrifugal concentrator to obtain treated CSs, which were then stored at -80 °C and used when applicable.

### Adherence assay

This was performed as described previously^3^. Briefly, HT29 cells were seeded at a density of 1 × 10^6^ cells/well in a 24 well plate and allowed to attach overnight. Cells were incubated with bacteria at a multiplicity of infection (MOI) of 10 in the absence or presence of CSs (diluted to 1x) for 1 h at 37 °C with 5% CO_2_. Cells were washed thrice in PBS to remove unattached bacteria, lysed with 1 mL of 0.01% Triton X-100 (Sigma) and dilution plated onto BHI or TSB agar plates. Adherence was calculated as the percentage of adhered bacteria vs. total bacteria added.

### Cell proliferation assay

This was performed as described previously^3^ with slight modifications. Briefly, cells were seeded in 96 well plates at a concentration of ~ 1 × 10^4^ cells/well and incubated overnight. Cells were then incubated in fresh DMEM/F-12 containing 10% FBS in the absence or presence of *Sgg* bacteria (MOI = 1) or *Sgg* CSs (1x) for a total of 24 h. Trimethoprim was added after 6 h of incubation (1 μg/mL final concentration) to inhibit bacterial growth, as previously described. The number of viable cells was determined using the cell counting kit (CCK)-8 kit following the instructions of the supplier (Apex Bio).

### Western blot

Detection of β-catenin and PCNA was carried out as described previously^3^. Briefly, HT29 cells were seeded at a density of 1 × 10^6^ cells per well in 6-well plates and incubated overnight. The cells were then incubated with media only or media containing bacteria (MOI = 1) for 9 h. Total cell lysates were subjected to SDS-PAGE, transferred, and probed with antibodies against β-catenin (1:1000), PCNA (1:1000), and β-actin (1:1000) (Cell Signaling Technology), followed by incubation with HRP-conjugated secondary antibodies (1:3000). Band intensities were measured using FIJI ImageJ and normalized to those of β-actin.

### Deletion of* SPAR*

This was performed following a procedure described previously^3,21^. Briefly, the ~ 1 kb region upstream of *sparA* and the ~ 1 kb region downstream of *sparL* were synthesized by Genscript and cloned into pUC57 (Genscript). The insert was then subcloned into a temperature sensitive conjugative plasmid pG1-*oriT*_TnGBS1_. The insert sequence was verified by Sanger sequencing. The construct was introduced into *S*. *agalactiae* NEM316 by electroporation and then into *Sgg* strain TX20005 by conjugation under the permissive temperature. PCR was used to screen for double cross-over deletion mutants. Deletion was further confirmed by PCR amplification of the regions spanning the deleted fragment and DNA sequencing of the PCR product. The genome of the parent strain TX20005 and TX20005∆*SPAR* was further sequenced by whole-genome shot-gun sequencing as previously described^3^. Deletion of the *SPAR* locus was validated in TX20005∆*SPAR*. The only other difference identified between the parent and the mutant strains was a 6 base pair insertion (CTCTGC) in an intergenic region around position 2,181,770 in TX20005∆*SPAR.* We performed RT-PCR to determine if the expression of the genes flanking the insertion site (*Sgg_2107* and *Sgg_*2108) was affected (Fig. S1). Results from RT-PCR indicated that this insertion had no effect on the expression of *Sgg_2107* or *Sgg_2108*. Thus, deletion of the *SPAR* locus is the only relevant mutation in TX20005∆*SPAR*. We next performed RT-PCR to determine if *SPAR* deletion affected the expression of the genes up and downstream of the *SPAR* locus, *Sgg_1056* and *Sgg_1070* (Fig. S2). These results indicated that the expression of these genes are not affected by *SPAR* deletion.

### RNA extraction, cDNA synthesis and PCR

This was performed following the method described previously^3^. Briefly, RNA was extracted from TX20005 cultured in the presence of HT29, as well as from the colonic tissues of mice that had been orally gavaged with TX20005. RNA was treated with DNase and synthesized using a ProtoScript II First Strand cDNA Synthesis Kit (NEB). Primers used in PCR amplification are: *sparA*, forward 5′GCAAGCTGGTCGAACAGAAC and reverse 5′GCTTCTATGGTTGGGGCTAGA; *sparD*, forward 5′GGAGGTGGATCCAACAAGGG and reverse 5′CAGGTTCCTCGATAGCCAGC; *sparG*, forward 5′TCAGTTGTTAGCGGATGCGT and reverse 5′ CCCTTTATTGCTTGTGCTCCC; *Sgg_1056*, forward 5′GCCGCACATGATTTCAGGG and reverse 5′GGGGACGCCGATAAGCC; *Sgg_1070,* forward 5′CCCTGCCAAAGCTGGCGG and reverse 5′GCCTTGCTTGTGACAAACCGTCATC; *Sgg_2107,* forward 5′CGCGAATGGTAAGGAATATCAAACTG and reverse 5′CCCACTAATACCTTTTCCACCTG; and *Sgg_2108,* forward 5′GCTGGCGTCTCGCGG and reverse 5′CCTGGGATGGAACTAAAAAGCAGTGC.

### Growth curves

Overnight cultures of *Sgg* were inoculated into fresh brain heart infusion (BHI) broth, DMEM/F-12 supplemented with 10% FBS, or conditioned media from HT29 or HCT116 cells cultured in DMEM/F-12 supplemented with 10% FBS at 1:100 dilution and grown at 37 °C with shaking. Optical density at 590 nm was determined at 0, 6, 12, and 24 h.

### Animal experiments

Animal studies were performed in accordance with protocols approved by the Institutional Animal Care and Use Committee at the Texas A&M Health Science Center, Institute of Biosciences and Technology. Mice were fed with standard ProLab IsoPro RMH3000 (LabDiet).

#### Colonization

This was performed as previously described^3^ with slight modifications. Briefly, 6-week-old A/J mice, sex matched (Jackson Laboratory), were treated with ampicillin at a concentration of 1 g/L in drinking water for 3 days and switched to antibiotic-free water 24 h prior to the administration of bacteria. *Sgg* was orally gavaged at a dose of ~ 1 × 10^9^ CFU/mouse. Colons and fecal materials were collected at day 1, 3, and 7 post-gavage. Samples were weighed, homogenized in sterile PBS in a TissueLyser (Qiagen), dilution plated onto Enterococcus Selective Agar (ESA) plates and incubated at 37 °C for 24–48 h to enumerate *Sgg* colonies.

#### AOM-induced model of CRC

This was performed as previously described^3^ with slight modifications. Briefly, 6-week-old A/J mice, sex matched were treated with 4 weekly i.p. injections of AOM (10 mg/kg body weight), followed by ampicillin in drinking water (1 g/L) for 7 days and switched to antibiotic-free water 24 h prior to the first oral gavage with *Sgg*. Mice were orally gavaged with bacteria at ~ 1 × 10^9^ CFU/mouse or saline (n = 11–12 per group) once per week for 12 weeks. Mice were euthanized one week after the last oral gavage by CO_2_ inhalation followed by cervical dislocation. Colon and fecal pellets were collected. The number and size of macroscopic tumor were recorded. Visual evaluation of colons was carried out by a blinded observer. A random subset of fecal pellets were weighed, homogenized in sterile PBS and dilution plated onto ESA plates.

### Statistical analysis

GraphPad Prism 9 was used for statistical analyses. Two-tailed unpaired *t*-test was used for pairwise comparisons to assess the significance of differences between two groups in cell proliferation assays, western blot analysis, and adherence assays. The non-parametric Mann–Whitney test was used to assess the significance of differences of results between groups in animal studies. Two-way ANOVA was used to analyze the bacterial growth curves. Ns, not significant, *p* ≥ 0.05; *p < 0.05; **p < 0.01; ***p < 0.001; ****p < 0.0001.

### Ethics statement

Animal studies were performed in accordance with protocols (IACUC#2017-0420-IBT) approved by the Institutional Animal Care and Use Committee at the Texas A&M Health Science Center, Institute of Biosciences and Technology. The Texas A&M University Health Science Center—Institute of Biosciences and Technology is registered with the Office of Laboratory Animal Welfare per Assurance A4012-01. It is guided by the PHS Policy on Human Care and Use of Laboratory Animals (Policy), as well as all applicable provisions of the Animal Welfare Act. This study is reported in accordance with ARRIVE guidelines.

## Results

### The *Sgg SPAR* locus

**Figure 1:**
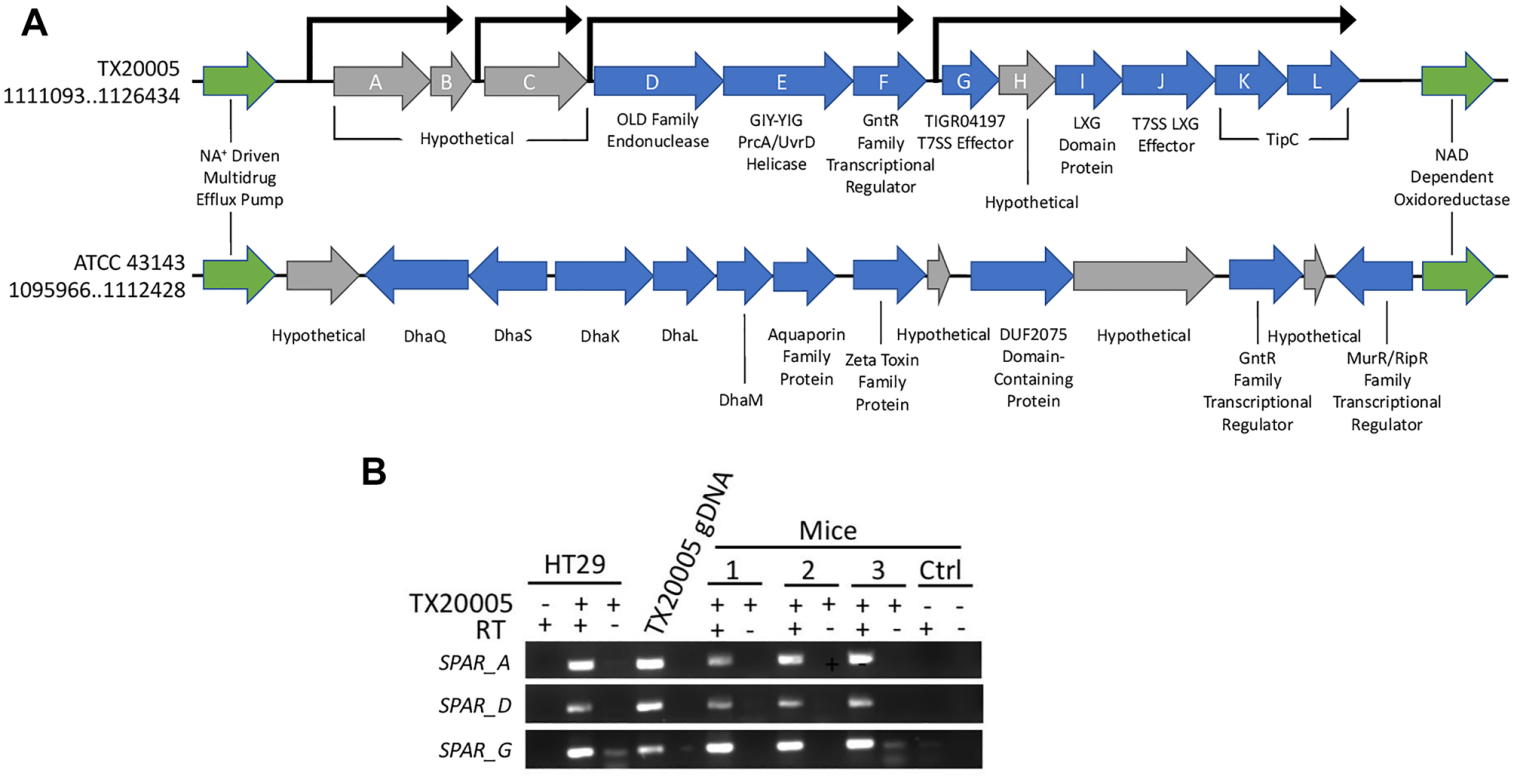
*SPAR* genetic organization and expression. (**A**) The *SPAR* locus. Genes encoding proteins with homology to proteins of known functions or containing characteristics of certain protein families are colored in blue, while genes encoding hypothetical proteins of unknown function are colored in gray. Comparable regions in TX20005 and ATCC 43143 are represented with genetic coordinates. (**B**) RT-PCR. cDNA synthesized from RNA extracted from HT29 cells co-cultured with TX20005 and the colonic tissues of three mice that had been orally gavaged with TX20005 were used as a template for the RT-PCR. cDNA from HT29 cells only and from mice orally gavaged with saline were used as controls to show the specificity of the PCR primers. RNA samples that had not been treated with reverse transcriptase (RT) were used as controls to show the lack of DNA contamination.

We compared the genome sequence of TX20005 (NZ_CP077423.1) with that of ATCC_43143 (NC_017576.1), an *Sgg* strain that is defective in stimulating CRC cell proliferation or promoting the development of colon tumors, using the multiple genome alignment tool MAUVE^22^. The *SPAR* locus is one of the regions in the chromosome of TX20005 that display differences from ATCC 43143 (Fig. 1A). The locus is comprised of 12 genes (*sparA* to *sparL*). Based on the genetic organization and results from FGENESB: Bacterial Operon and Gene Prediction tool (softberry), the 12 genes are organized into three putative operons; *sparAB, sparD-F, sparG-L,* and 1 standalone gene, *sparC*. The protein sequences encoded by these genes were analyzed for secretion signals using SignalP-6.0^23^. None of them contains typical secretion signals recognized by the Sec or Tat translocon. SparA, SparK and SparL are predicted to contain transmembrane helices (TMHMM2.0)^24^ and are thus putative transmembrane proteins. Homology search using Protein BLAST showed that SparA to C are hypothetical proteins of unknown function (Table 1). SparD to SparF exhibit homology to predicted ATP-dependent endonuclease of the OLD family, GIY-YIG endonuclease/PcrA/UvrD helicase, and GntR family transcriptional regulator, respectively. SparG to SparJ display features of effectors secreted by the type VII secretion system (T7SS). SparG, H and I are of 91, 102 and 137 amino acids in length, in keeping with T7SS effectors belonging to the WXG100 family^25^. Analysis using HHpred^26^ showed that SparG and SparI fold into a four-helical bundle structure typical of WXG100 proteins, with a probability of 96.48% and 97.97%, respectively. Spar H and I also contain a conserved sequence motif HxxxD/ExxhxxxH (H denotes highly conserved and h less conserved hydrophobic residues) that is considered to be important for the secretion of WXG100 proteins by T7SS^27^ (Supplemental Fig. S3). Based on these sequence and structural characteristics, SparG, H and I are likely WXG100 proteins (Table 1). Protein BLAST search showed that SparJ belongs to the T7SS effector LXG polymorphic toxin family^28,29^. The N-terminal portion of SparJ contains a centrally located LXG motif as well as the HxxxD/ExxhxxxH motif (Supplemental Fig. S3), features consistent with an LXG toxin. Immediately downstream of the *sparJ* gene are two genes that encode putative TipC family immunity proteins. Genes encoding T7SS effector LXG polymorphic toxins are typically clustered together with genes encoding their cognate immunity proteins^28–30^. The arrangement of *sparJ*, *K* and *L* provides further support that SparJ is a putative T7SS effector LXG polymorphic toxin and SparK and L are likely the cognate immunity proteins.

**Table 1:** Proteins encoded by the *SPAR* genes.

Gene	Coordinates^a^	Length (aa)^a^	Predicted gene function^b^	Prevalence(%)^d^
*sparA*	1112509..1113783	424	Hypothetical protein	4/9 (44)
*sparB*	1113840..1114001	53	Hypothetical protein	8/9 (89)
*sparC*	1114145..1115893	582	Hypothetical protein	5/9 (56)
*sparD*	1116106..1118076	656	OLD-Family endonuclease	4/9 (44)
*sparE*	1118073..1119902	609	GIY-YIG Endonuclease/PcrA/UvrD Helicase	8/9 (89)
*sparF*	1119965..1120663	232	GntR-Family Transcriptional regulator	8/9 (89)
*sparG*	1120767..1121042	91	WXG100 family protein^c^	8/9 (89)
*sparH*	1121044..1121352	102	WXG100 family protein^c^	6/9 (67)
*sparI*	1121342..1121755	137	WXG100 family protein^c^	7/9 (78)
*sparJ*	1121774..1123027	417	T7SS effector LXG polymorphic toxin	7/9 (78)
*sparK*	1123011..1123610	199	TipC family immunity protein	6/9 (67)
*sparL*	1123657..1124250	197	TipC family immunity protein	7/9 (78)

^a^Based on the annotated genome of TX20005 (NZ_CP077423.1).

^b^The amino acid sequence of each protein was used to search the Non-redundant protein sequences database (updated on 04/05/2022) at NCBI using Protein BLAST.

^c^Based on characteristic sequence motifs and structural fold predictions displaying features of WXG100 proteins.

^d^The amino acid sequence of each protein was used to search the nine complete *Sgg* genomes at NCBI using BLAST Genomes, optimized for highly similar sequences (megablast). The number of genomes containing highly similar sequences vs. the total number of genomes searched are shown. The numbers in the parenthesis indicate the percentage of positive genomes.

To determine the prevalence of the *SPAR* genes among *Sgg* strains, we performed BLAST Genomes search of the nine complete *Sgg* genomes at NCBI with each of the encoded proteins. The results showed that the prevalence varies from 44% (4 of 9 strains) to 89% (8 of 9 strains) for the different genes (Table 1). We also examined if orthologs of these genes are present in other SBSEC members, *Streptococcus gallolyticus* subspecies *macedonicus* (*SGM;* taxid: 59310) and subspecies *pasteurianus* (*SGP;* taxid: 197614)*,* as well as *Streptococcus infantarius* subsp. *infantarius* (*SII;* taxid: 150054)*.* SparA, and H–L are absent in these other members, suggesting that they are unique to *Sgg* within the SBSEC members. SparB and C are present in *SGP* but not in other members, and SparG is present in *SGM* but not others. However, these species all contain orthologs of SparB, E and F. The varied prevalence of the *SPAR* genes among *Sgg* strains and species within the SBSEC suggest that the *SPAR* region likely did not arise from a recent acquisition of a mobile genetic element.

We next tested if the *SPAR* genes were expressed in vitro and in vivo by RT-PCR (Fig. 1B). We focused on the expression of *sparA*, *D* and *G*, under conditions that are relevant to the pro-proliferation and pro-tumor activity of *Sgg*. All three genes were expressed when the bacteria were co-cultured with HT29 cells and in the colonic tissues collected from mice orally gavaged with TX20005 (Fig. 1B).

### *SPAR *is important for *Sgg* adherence

**Figure 2:**
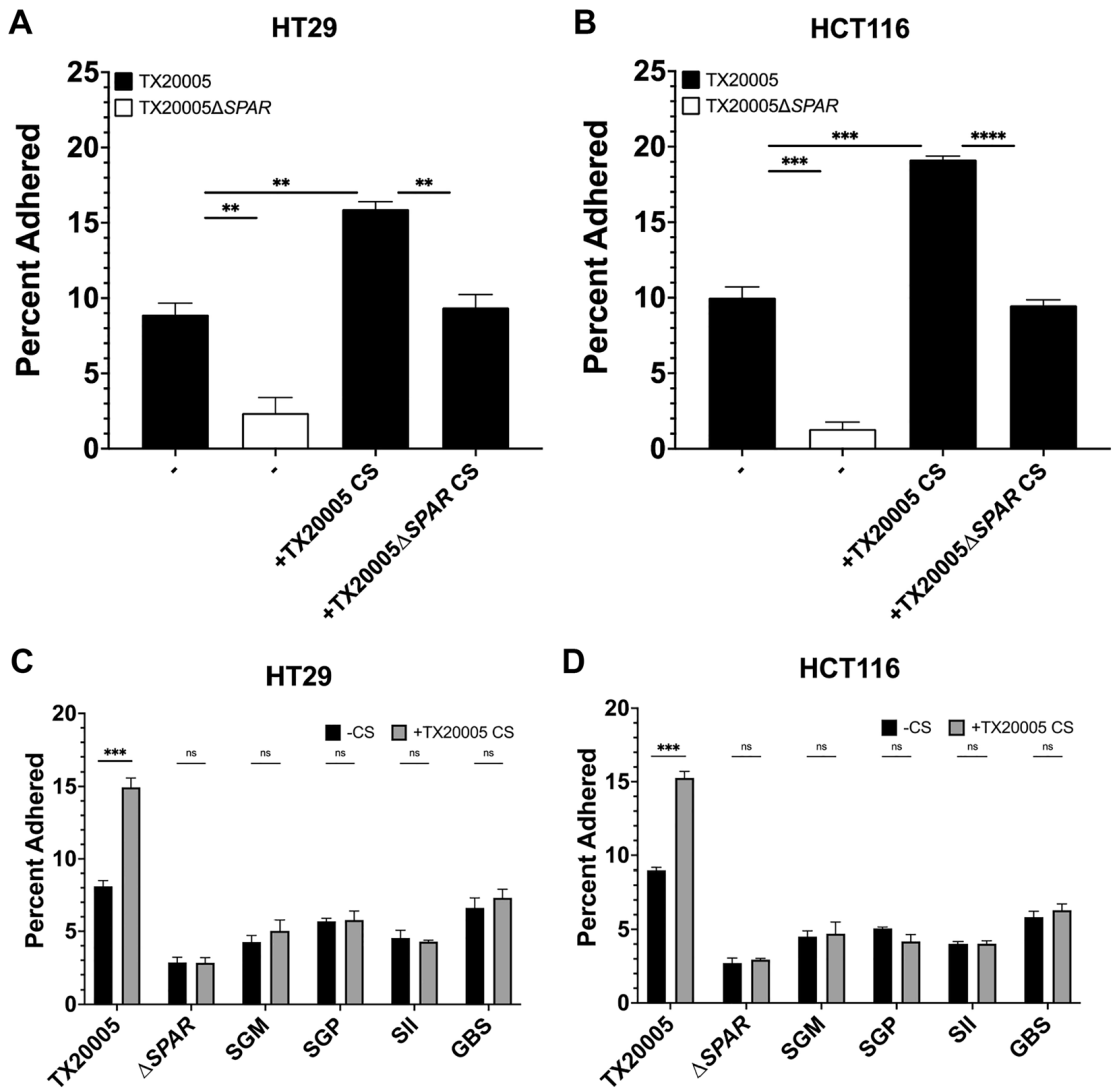
*SPAR* is important for *Sgg* adherence to host cells. HT29 (**A**) and HCT 116 (**B**) cells were seeded at a density of ~1 × 10^6^ cells per well and incubated with TX20005 or TX20005*ΔSPAR* (MOI = 10) in media only or in the presence of CS from TX20005 or TX20005*ΔSPAR* for 1 h. HT29 (**C)** and HCT 116 (**D**) cells were seeded at a density of 1 × 10^6^ cells per well and incubated with various bacteria (MOI = 10) for 1 h. SGM, *Streptococcus gallolyticus* subspecies *macedonicus*; SGP, *Streptococcus gallolyticus* subspecies *pasteurianus*; SII, *Streptococcus infantarius* subsp. *infantarius*; GBS, group B streptococcus. Percentage adherence was calculated as the percentage of adhered bacteria vs total bacteria added. Results were combined from three biological replicates. Unpaired two-tailed *t* tests were used. **p < 0.01; ***p < 0.001; NS, not significant. The mean + /− SEM are plotted.

To investigate the role of the *SPAR* locus in the pathogenic potential of *Sgg*, we generated a mutant in the TX20005 background, in which all 12 genes were deleted (TX20005∆*SPAR*). We first verified that *SPAR* deletion does not affect bacterial growth. To determine this, we compared the growth of the wild type (WT) and the mutant in BHI broth (Fig. S4A), DMEM/F-12 supplemented with 10% FBS (Fig. S4B), and conditioned media from either HT29 (Fig. S4C) or HCT116 cells cultured in DMEM/F-12 with 10% FBS (Fig. S4D). The results indicate that the mutant showed similar growth patterns compared to the parent strain in all three conditions tested. We next sought to determine if deletion of the *SPAR* locus had any effect on the adherence capacity of *Sgg*. The results showed that the mutant strain exhibited a significantly reduced adherence to HT29 cells compared to the WT parent strain TX20005 (~ 2% vs ~ 9%; *p* < 0.01) (Fig. 2A). This phenotype was also observed in HCT116 cells (~ 1% vs ~ 10%; *p* < 0.001) (Fig. 2B), suggesting that the *SPAR* locus is important for *Sgg* adherence to these cells.

Previous work showed that T7SS-secreted factors of *Sgg* significantly enhanced the adherence capacity of TX20005^3^. We tested whether filter sterilized culture supernatants (CS) from TX20005Δ*SPAR* can enhance the adherence capacity of TX20005. Addition of CS from the WT TX20005 significantly enhanced the adherence of WT bacteria (*p* < 0.01) as expected, whereas addition of the mutant CS did not cause any increase in the adherence of WT bacteria to HT29 cells (Fig. 2A; *p* < 0.01). This phenotype was also observed in HCT116 cells (Fig. 2B; *p* < 0.001), suggesting that *SPAR*-dependent secreted factors contribute to *Sgg* adherence. Previous work also indicated that the WT CS is insufficient to restore the defective adherence phenotype of a T7SS mutant, and that T7SS-dependent surface anchored factors are also needed for adherence. We tested the effect of CS on the adherence capability of TX20005Δ*SPAR*. The results showed that neither WT CS or the TX20005Δ*SPAR* CS was able to restore the adherence phenotype of TX20005∆*SPAR* with HT29 (Fig. S5A) or HCT116 cells (Fig. S5B). This result is similar to the previous observations and suggests that secreted factors alone are insufficient to mediate *Sgg* adherence.

We next tested the ability for the WT CS to enhance the adherence of other closely related streptococcal species: SGM, SGP, SII, and *S. agalactiae* NEM316 (group B streptococcus (GBS)). Adherence was performed in the presence or absence of TX20005 CS with HT29 (Fig. 2C) and HCT116 cells (Fig. 2D). These results demonstrated that enhanced adherence was only exhibited in WT TX20005 (HT29, p < 0.001; HCT116, *p* < 0.001), and no increase in adherence was observed in SGM, SGP, SIC, or GBS. Taken together, these results indicate that *SPAR* is important for the adherence capacity of *Sgg* and suggests that both *SPAR*-dependent secreted and surface anchored factors are involved in *Sgg* adherence. Moreover, these results demonstrated a species level specificity for this phenotype.

### *SPAR* is required for *Sgg* to stimulate CRC cell proliferation

**Figure 3:**
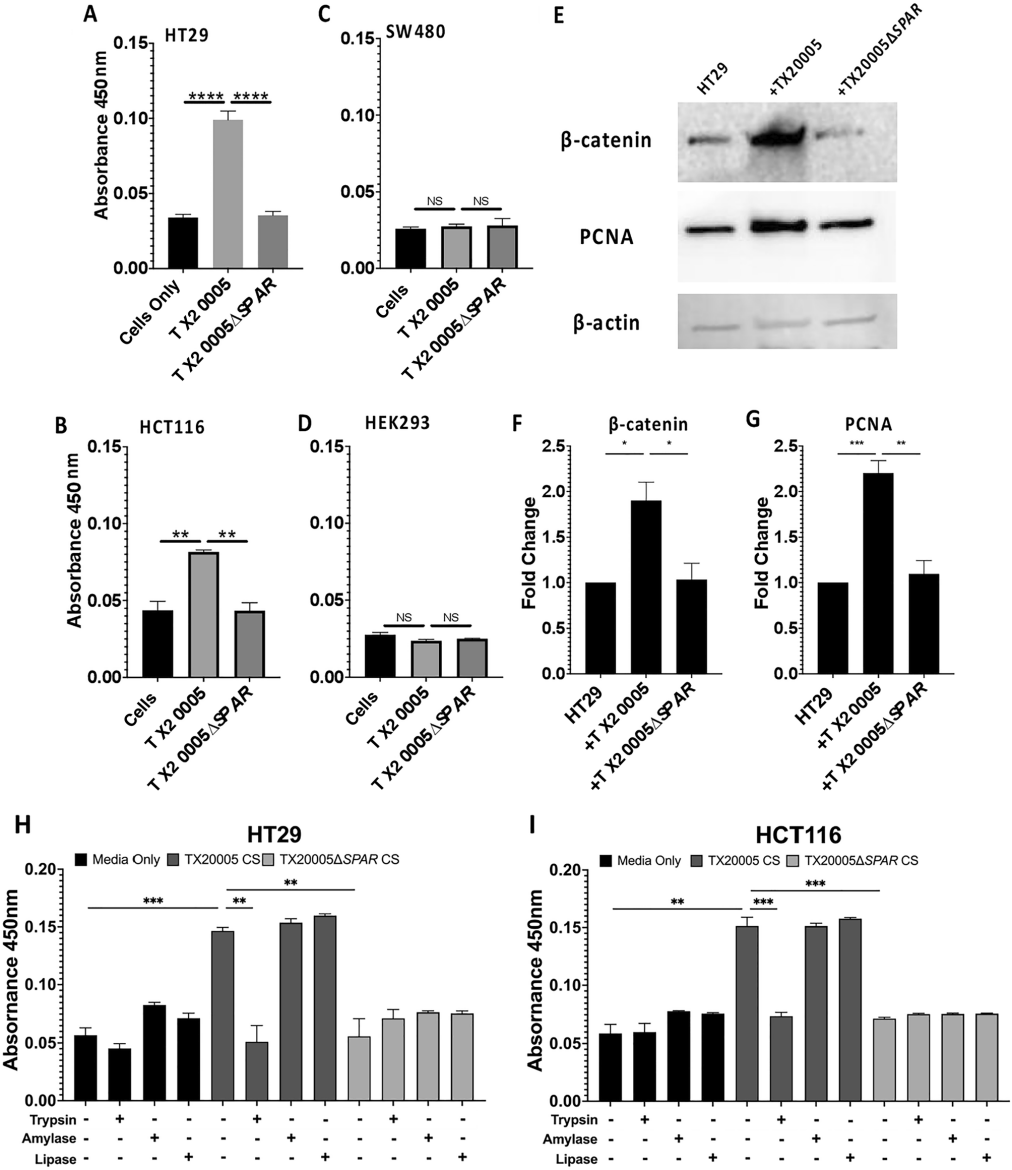
*SPAR* is essential for *Sgg* to stimulate CRC cell proliferation. **A**-**D**. Cell proliferation assay. HT29 (**A**), HCT116 (**B**), SW480 (**C**), and HEK293 (**D**) cells were incubated in the presence or absence of bacteria (MOI = 1) or CSs for 24 h, as described in the “Materials and Methods” section. Cell proliferation was measured using a CCK-8 kit. Cell-free wells containing only the culture media served as a blank control to which the absorbance values were normalized. **E**–**G**. Western blot. Cell lysates from HT29 cells co-cultured in the presence or absence of bacteria (MOI = 1) were analyzed by western blot, probed with antibodies against β-catenin, PCNA, and β-actin (**E**). Band intensity was quantified and normalized to β-actin. Fold change in β-catenin (**F**) and PCNA (**G**) were calculated against cells incubated in media only. Data was combined from three biological replicates. **H** and **I**. Cell proliferation in the presence of *Sgg* CSs. HT29 cells (**H**) or HCT116 cells (**I**) were cultured in the presence or absence of WT or mutant *Sgg* (MOI = 1) or CSs from WT or mutant *Sgg* for 24 h that were untreated with any enzyme, or pretreated with 50 mM trypsin, 50 mM α-amylase, or 50 mM lipase for 1 h at 37 °C. Cell proliferation was measured by using a CCK-8 kit. Results were combined from at least three biological replicates. Unpaired two-tailed *t* tests were used for the comparisons. *p < 0.05; **p < 0.01; ***p < 0.001; ****p < 0.0001; ns, not significant. Significance in panels H and I indicates comparison to untreated cells. Results are combined from at least three independent experiments. The mean + /− SEM are plotted.

*Sgg* has been previously shown to stimulate cell proliferation in human CRC cell lines HT29 and HCT116, but not in SW480 or HEK293 cell lines^2–4^. We examined the ability of TX20005∆*SPAR* to stimulate cell proliferation in these cell lines. HT29, HCT116, SW480, and HEK293 cells were co-cultured with TX20005 or TX20005∆*SPAR*. As anticipated, the WT strain significantly stimulated HT29 and HCT116 cell proliferation compared to untreated cells (Fig. 3A,B; *p* < 0.0001 and *p* < 0.01, respectively; and Fig. S6). In contrast, HT29 and HCT116 cells co-cultured with the mutant did not exhibit any significant increase in cell proliferation, indicating that *SPAR* is required for *Sgg*-stimulated CRC cell proliferation. Neither the WT nor the mutant had any effect on the proliferation of SW480 or HEK293 cells (Fig. 3C,D), as expected.

To further validate the results, we investigated the effect of the mutant on cell proliferation markers β-catenin and proliferating cell nuclear antigen (PCNA) in HT29 cells (Fig. 3E–G). Cells co-cultured with TX20005 exhibited a significant increase in the level of both β-catenin (*p* < 0.05) and PCNA (*p* < 0.001) compared to cells cultured in media only, as expected. In contrast, cells co-cultured with the *SPAR* mutant showed no significant increase in the level of either marker compared to cells cultured in media only, further confirming that the *SPAR* locus is essential for *Sgg* to stimulate cell proliferation.

Previous work demonstrated that filter sterilized CSs from WT *Sgg* strains were able to induce CRC cell proliferation^3^. We examined the effect of CS from TX20005∆*SPAR* on cell proliferation (Fig. 3H,I). HT29 cells treated with the WT CS exhibited a significant increase in cell proliferation compared to untreated cells (*p* < 0.001; Fig. 3H), as expected, whereas cells treated with the mutant CS did not exhibit any significant increase. Similar results were observed in HCT116 cells (Fig. 3I). These results suggest that the defect in the *SPAR* mutant to stimulate host cell proliferation is due, at least in part, to the absence of certain secreted factors in the CS. To ascertain the nature of the active molecules in the CS that are responsible for stimulating cell proliferation, we treated the CSs with trypsin, α-amylase, or lipase. Trypsin treatment of WT CS abrogated its ability to promote cell proliferation in HT29 (*p* < 0.01 vs untreated CS; Fig. 3H) and HCT116 cells (*p* < 0.001 vs untreated CS; Fig. 3I), whereas α-amylase or lipase treatment did not prevent the WT CS from stimulating HT29 or HCT116 cell proliferation, suggesting that the active molecules in the CS are proteinaceous in nature. Taken together, these results indicate that *SPAR*, and particularly *SPAR*-dependent secreted protein(s), is required for *Sgg* to stimulate CRC cell proliferation in vitro.

### *Sgg *gut colonization is impaired by *SPAR* deletion

**Figure 4:**
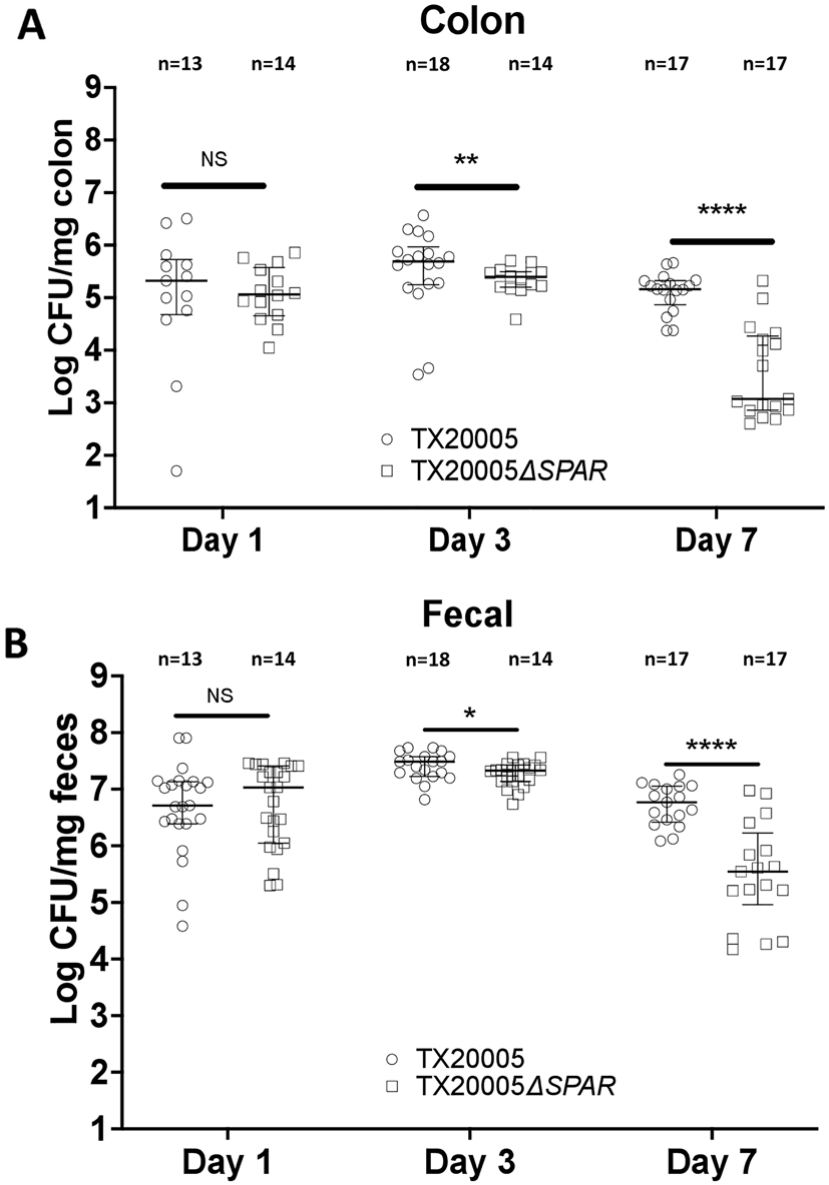
*SPAR* deletion reduced the colonization capacity of *Sgg*. This was performed as described in the “Materials and Methods” Section. 13–18 mice were used per group. Colon tissues (**A**) and fecal pellets (**B**) were weighed, homogenized in sterile PBS and dilution plated onto ESA plates. Mann–Whitney test was used for statistical analysis. *, *p* < 0.05; **, *p* < 0.01; ****, *p* < 0.0001; NS, not significant. Median values with interquartile range (IQR) are plotted.

The ability of *Sgg* to colonize the host gut is an important aspect of its pathogenic potential^2^. We sought to determine if the *SPAR* locus is involved in *Sgg* gut colonization. Mice were orally gavaged with WT or the *SPAR* deletion mutant. Colon tissues as well as fecal pellets were collected at day 1, 3, and 7 post-bacterial gavage to determine the bacterial load in the samples. At day 1, the *Sgg* bacterial load in the colonic tissues from mice gavage with the WT and the mutant did not significantly differ. At day 3 and 7, the bacterial load of the mutant in the colonic tissues was significantly decreased compared to that of the WT (Fig. 4A; ~ 67% reduction of the mean, *p* < 0.01, day 3 and ~ 99% reduction of the mean, *p* < 0.0001, day 7). In the fecal pellets, a similar patten was observed, such that at day 1, the bacterial load of mutant and WT strains did not significantly differ, while at day 3 and 7, the bacterial load of the mutant was decreased significantly compared to that of the WT (Fig. 4B; ~ 33% reduction of the mean, *p* < 0.05, day 3 and ~ 95% reduction of the mean, *p* < 0.0001, day 7). Taken together, these results indicate that the *SPAR* locus contributes to the colonization capacity of *Sgg* in the normal colon.

### *SPAR* is essential for the ability of *Sgg* to promote the development of colon tumors

**Figure 5:**
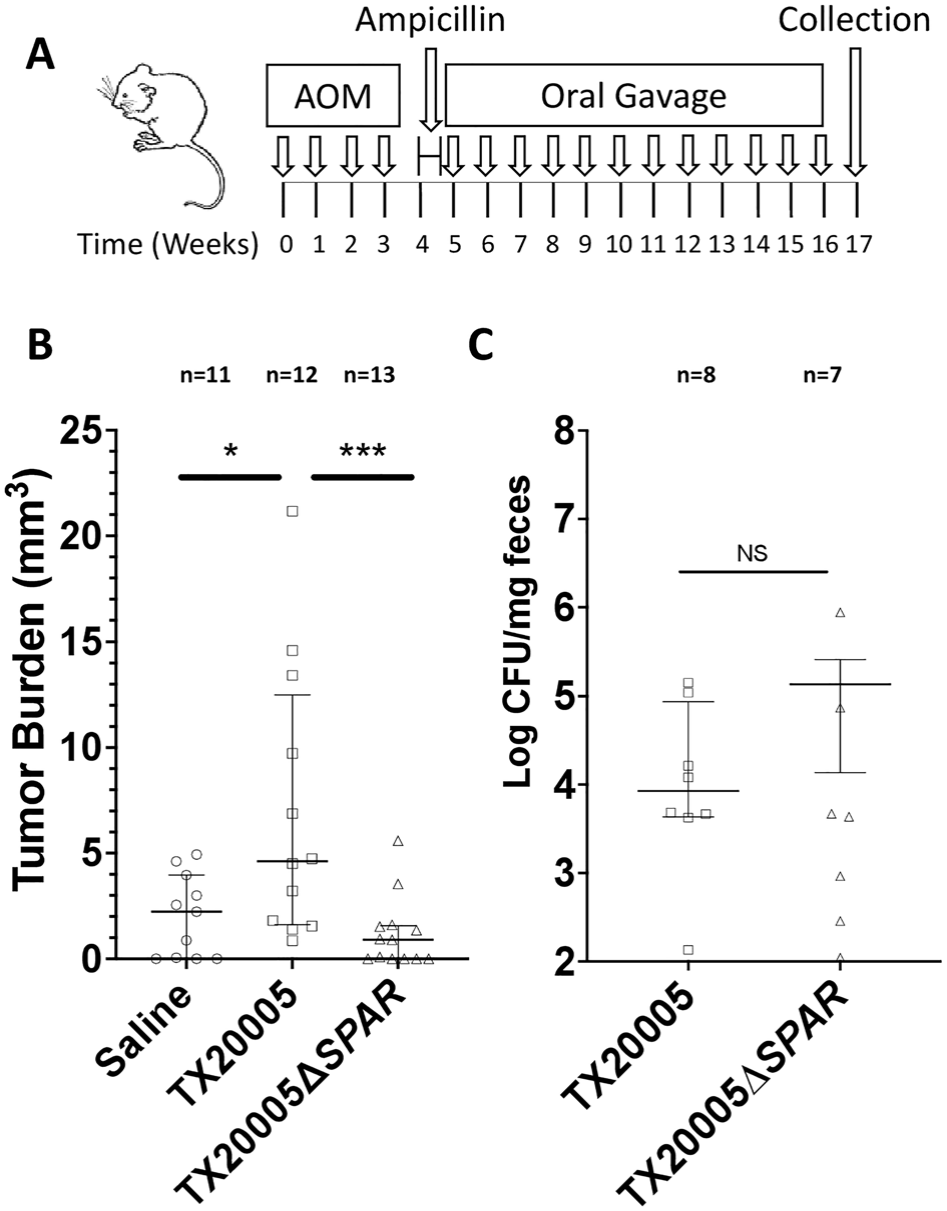
*SPAR* is critical for *Sgg* to promote the development of colon tumors. This was performed as described in the “Materials and Methods” section. The procedure is illustrated in (**A**). (**B**) The number and size of macroscopic colon tumors were recorded by blinded observers. Tumor burden is calculated as the sum of tumor volumes per mouse. 11–13 mice were used per group. (**C**) *Sgg* load in the fecal pellets collected at the experimental endpoint was determined by dilution plating of homogenized pellets onto ESA plates. 7 or 8 mice per group were used. Mann–Whitney test was used for statistical analysis. *p < 0.05; ***p < 0.001; NS, not significant. Median values with interquartile range (IQR) are plotted.

Next, we investigated if *SPAR* is important for *Sgg* to promote the development of colon tumors using an AOM-induced CRC model (Fig. 5A), as described previously^3^. Mice gavaged with the WT strain exhibited a significant increase in the colon tumor burden (Fig. 5B; *p* < 0.05) compared to saline-gavaged mice, consistent with previous results^3,4^. In contrast, mice exposed to the *SPAR* mutant showed no increase in the tumor burden compared to the saline control and a significant reduction compared to the WT-treated mice (*p* < 0.001), suggesting that *SPAR* is important for *Sgg* to promote colon tumors. We also determined the bacterial load in the fecal pellets isolated at the end of the experiment and found no difference in the *Sgg* burden between WT or mutant-treated groups (Fig. 5C). We note that this result could be due to the repeated gavages of bacteria during the experiment and does not necessarily reflect the ability of the strains to colonize tumor-bearing colons in this model. These results together suggest that *SPAR* plays a critical functional role in promoting the development of colon tumors.

## Discussion

In this study, we describe an *Sgg* chromosomal locus, *SPAR*, that plays a key role in *Sgg* pathogenesis. Deletion of *SPAR* led to striking phenotypes in vitro and in vivo, including a reduced capacity to adhere to CRC cells, decreased colonization of the normal colon, and a complete loss of the ability to stimulate CRC cell proliferation in vitro and to promote the development of colon tumors in vivo. These results highlight *SPAR* as a critical pathogenicity determinant of *Sgg*.

Our data showed that deletion of the *SPAR* locus resulted in significantly reduced adherence of *Sgg* TX20005 to HT29 and HCT116 cells. Our previous study showed that T7SS-secreted factors enhance TX20005 adherence^3^. Interestingly, our results revealed that deletion of *SPAR* eliminated the adherence-enhancing activity in the CS, suggesting that the deletion might have affected the T7-secreted factors involved in adherence. In terms of cell proliferation, we showed that the *SPAR* locus is essential for TX20005 to stimulate certain CRC cell proliferation. Furthermore, the results indicate that *SPAR* is also important for the production of secreted factors responsible for stimulating host cell proliferation. In this regard, the *SPAR* mutant exhibits a very similar phenotype as that described for a T7SS defective mutant of *Sgg* (TX20005*Δesx*) with respect to adherence and stimulation of cell proliferation^3^. One possible reason for the phenotypic similarity is that the *SPAR* locus encodes several putative T7SS effectors (SparG to SparJ). Examination of the TX20005 genome reveals that the previously reported T7SS locus (*Sgg*T7SS^T05^)^3^ is the only locus that encodes a set of proteins comprising the T7SS secretion apparatus. Thus, SparG to SparJ are likely secreted by *Sgg*T7SS^T05^. This would imply that one or more of the *SPAR*-encoded putative T7SS effectors are involved in the adherence and the pro-proliferative activity of *Sgg*. On the other hand, the *sparF* gene is predicted to encode a GntR family transcriptional regulator^31^. A recent publication indicated that a GntR family transcriptional regulator (OG1RF_11099) of *Enterococcus faecalis* controls the expression of T7SS genes^32^. Using Global Align (NCBI), we found that SparF is highly homologous to OG1RF_11099, showing an overall 74% similarity at the amino acid sequence level. Hence, a second possible reason for the phenotypic similarity between TX20005*Δspar* and TX20005*Δesx* is that SparF regulates the expression of genes in the *Sgg*T7SS^T05^ locus. In this regard, we tested the effect of *SPAR* deletion on the expression of two genes in the *Sgg*T7SS^T05^ locus: *esxA*, the first gene in the locus, and *essC*, an essential component of the T7 secretion machinery. We could not detect the expression of either of these genes in the *SPAR* mutant (Fig. S7), supporting a role of *SPAR* in controlling *Sgg*T7SS^T05^ expression. Further studies to delineate the function of SparF will be important.

In a gut colonization model, we observed that deletion of *SPAR* resulted in reduced *Sgg* bacterial load in the normal colon. We noted a decrease of bacterial load from day 3 to 7 for both the WT and the mutant bacteria. This is consistent with previous results showing that after a single oral gavage, *Sgg* burden decreases over time in normal colons of A/J and C56BL/6 mice^2,3,33,34^. The reduced colonization capacity observed for the mutant may be due to several factors. First, SparJ is a putative T7SS-secreted polymorphic toxin. This family of toxins are often involved in interbacterial competition^28^. The likelihood of SparJ as an antibacterial toxin is further strengthened by the two immediate downstream genes which encode TipC family immunity proteins. In addition, the T7SS locus of TX20005 also encodes a putative polymorphic toxin, *Sgg*511^3^. The expression of *Sgg*511 may be controlled by SparF. Thus, the lack of SparJ and the transcription suppression of *Sgg*511 in the mutant may impair the capacity of the bacteria to antagonize other commensal bacteria in the gut. A reduced bacterial load of the mutant in the lumen can also lead to less bacteria available to attach to the colonic mucosa. Second, previous studies using immunofluorescence microscopy of colon sections from mice orally gavaged with *Sgg* or tumor biopsies from CRC patients showed that *Sgg* bacteria were visualized within tumor tissues in close contact with tumor cells^4^, suggesting that *Sgg* is able to penetrate the mucus layer and reach the surface of these cells. Thus, *SPAR* may also contribute to colonization of the colonic epithelium by facilitating *Sgg* adherence. Other *Sgg* factors have previously shown to mediate gut colonization or adherence to host tissues. The Pil3 pilus of *Sgg* binds to intestinal mucin^35^ and contributes to the colonization of the distal colon of C57BL/6 mice^36^. The Pil1 pilus mediates binding to collagen^37,38^ and may facilitate *Sgg* adherence to surface-exposed collagen in the colonic tissues. Gallocin, a bacteriocin secreted by *Sgg*, facilitates *Sgg* colonization of tumor-bearing colons by suppressing the growth of other commensal bacteria^34^. Whether deletion of *SPAR* affects the expression of these factors is currently unknown. However, the moderate reduction of the mutant bacterial load observed in this study suggests that other factors contribute to *Sgg* colonization in the absence of *SPAR*. It is possible that these other factors may be Pil3, Pil1, gallocin or additional factors that are yet to identified.

In the AOM model of CRC, mice exposed to TX20005 showed a significantly higher tumor burden compared to mice treated with saline, whereas mice gavaged with the *SPAR* mutant showed no difference in tumor burden compared to the saline control. Thus, the *SPAR* mutant has completely lost the ability to promote the development of colon tumors. Notably, TX20005∆*SPAR* bacterial burden in the colon is not significantly different from that of TX20005. These results, combined with the finding that *SPAR* deletion completely abolished the pro-proliferative activity of *Sgg*, suggest the *SPAR* locus plays a functional role in *Sgg’*s pro-proliferative and pro-tumor activity. The *SPAR* locus may contribute directly to this phenotype via some of the *SPAR* genes, or indirectly by regulating the expression of other genes important for promoting tumor development including T7SS or non-T7SS genes via SparF. Studies to further investigate the biological activities of SparF, and SparG to SparL are needed to delineate the specific contribution of the *SPAR* locus to the observed phenotype. We note that some of the mice exposed to TX20005 did not show any increase in tumor burden compared to the control mice, exhibiting a certain level of heterogeneity in the ability of TX20005 to promote tumor growth. Similar results have been observed in the AOM model previously^2,3^. TX20005 stimulates the proliferation of certain CRC cells but not others, displaying a context dependent effect. It is possible that different mutations induced by AOM impart different susceptibility to the effect of TX20005, resulting in the observed heterogeneity in the tumor burden. Whether the expression of the *SPAR* genes or *SPAR*-regulated genes is influenced by the different genetic context is unknown and may also contribute to the heterogeneity.

It is possible that other genes within the *SPAR* locus, SparA-SparE, also play a role, however, their predicted function based on homology search does not provide clues regarding their respective contribution. SparA-C are putative hypothetical proteins. SparD is homologous to OLD family endonucleases. OLD family endonucleases are widely present among bacteria and archaea^39^. While their biological function is not completely understood, they contain a C-terminal ATPase domain, as well as an N-terminal Toprim domain, which is believed to be important in DNA replication, recombination, and repair^40,41^. SparE is homologous to GIY-YIG endonuclease^42–46^ and PcrA/UvrD helicase. The PcrA/UvrD helicase has been shown to play a role in DNA repair, as well as the replication of small drug-resistance plasmids in *Staphylococcus aureus* and *Bacillus subtilis*^47–53^. Pinpointing which genes are responsible for the phenotypes observed in this study is critical to correlating genotype with phenotype.

We encountered difficulties in complementing the *SPAR* deletion mutant with either the entire *SPAR* fragment or segments containing the putative operons. This could be partly due to the technical challenge of introducing large segments of DNA into *Sgg*. Another possible factor is the putative function encoded by some of the genes (*e.g*., endonuclease, LXG toxin) causing damage to the cloning host. Alternative methods need to be explored to further dissect the function of the *SPAR* locus. In addition, given the essential role of *SPAR* in regulating the expression of genes within the *Sgg*T7SS^T05^ locus (Fig. S6), future studies to investigate the crosstalk between *SPAR* and *Sgg*T7SS^T05^ will be important.

In summary, we have identified a chromosomal locus in *Sgg* strain TX20005, *SPAR*, that is critical to the pathogenicity of *Sgg.* We report that deletion of *SPAR* significantly reduces the capacity of *Sgg* to adhere to CRC cells and to colonize the gut. Furthermore, deletion of *SPAR* abrogates the ability for *Sgg* to stimulate CRC cell proliferation or to accelerate colon tumor development. Examination of genes with the locus highlighted several candidates relevant to the observed phenotypes via direct involvement or indirectly by regulating the expression of other genes. The results also indicate a connection between the *SPAR* locus and the T7SS of *Sgg* in terms of additional T7SS effectors encoded by *SPAR* genes and a potential regulator of T7SS expression within the *SPAR* locus. Further investigations into the activities of specific *SPAR* proteins will be important for dissecting the intricate *Sgg* pathogenic mechanisms and will open up the path ahead to identify biomarker candidates and targets for clinical prevention and intervention strategies.

## Supplementary Information

Supplementary Information 1.

Supplementary Information 2.

Supplementary Information 3.

Supplementary Information 4.

Supplementary Information 5.

Supplementary Information 6.

Supplementary Information 7.

Supplementary Information 8.

## Data Availability

The datasets used and/or analyzed during the current study are available from the corresponding author on reasonable request.
