# Supplementary figures and images for: A pathogenicity locus of Streptococcus gallolyticus subspecies gallolyticus
Source: Sci Rep. 2023 Apr 18;13:6291. doi: 10.1038/s41598-023-33178-z (PMC10113328; doi:10.1038/s41598-023-33178-z)

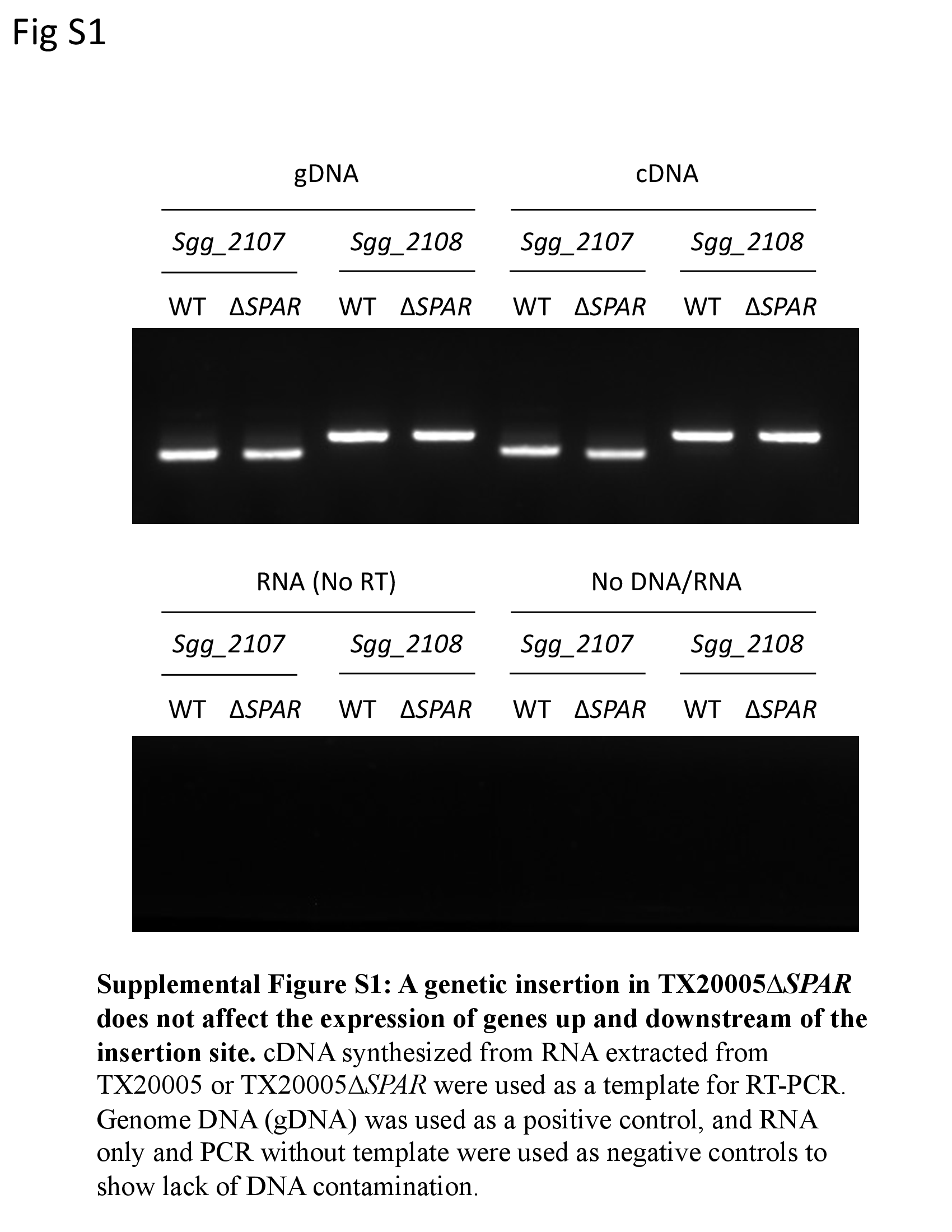

Supplement: Supplementary file 1 — Supplementary Information 1. [file 41598_2023_33178_MOESM1_ESM.tiff]

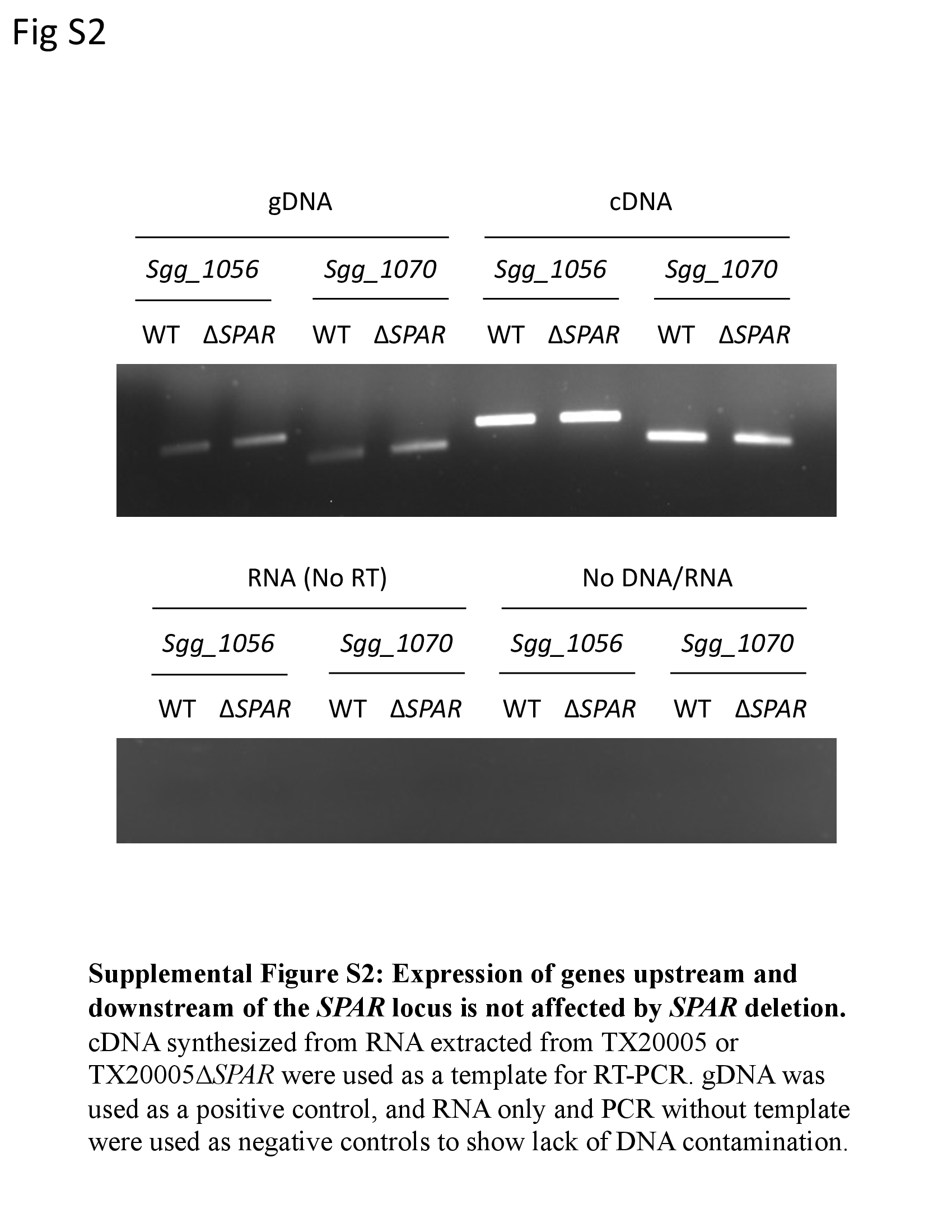

Supplement: Supplementary file 2 — Supplementary Information 2. [file 41598_2023_33178_MOESM2_ESM.tiff]

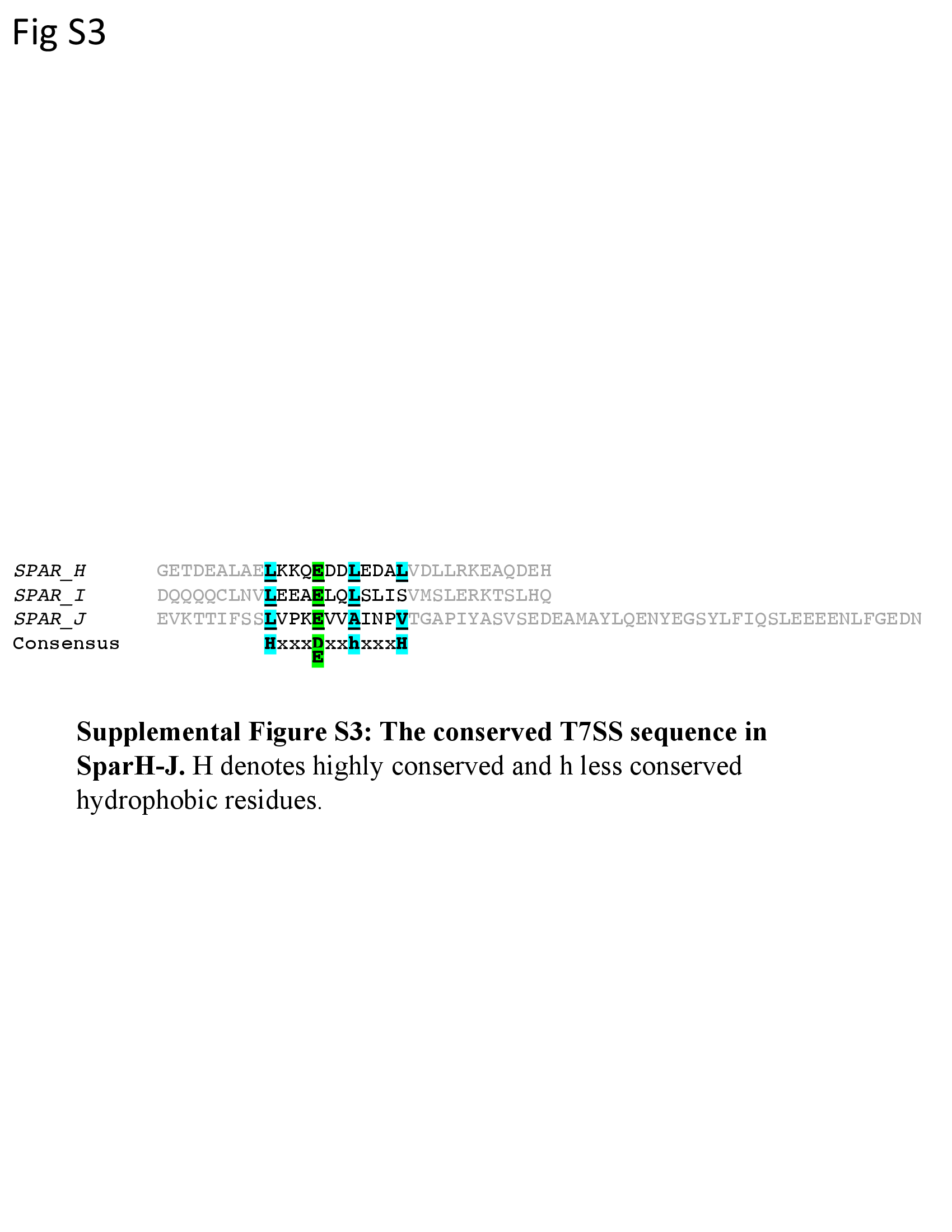

Supplement: Supplementary file 3 — Supplementary Information 3. [file 41598_2023_33178_MOESM3_ESM.tiff]

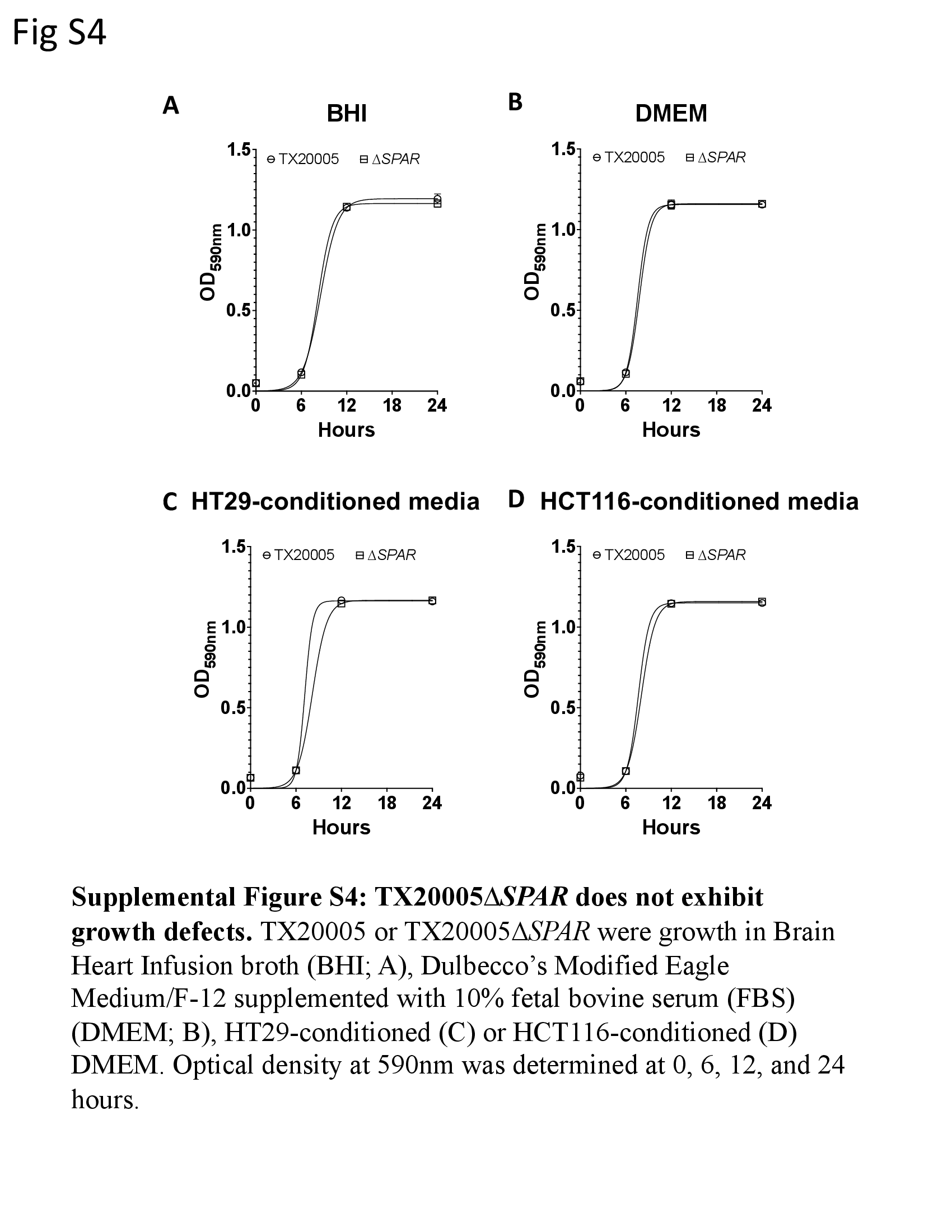

Supplement: Supplementary file 4 — Supplementary Information 4. [file 41598_2023_33178_MOESM4_ESM.tiff]
