# Supplementary material for: A pathogenicity locus of Streptococcus gallolyticus subspecies gallolyticus
Source: Sci Rep. 2023 Apr 18;13:6291. doi: 10.1038/s41598-023-33178-z (PMC10113328; doi:10.1038/s41598-023-33178-z)
Supplement: Supplementary file 8 — Supplementary Information 8. [file 41598_2023_33178_MOESM8_ESM.pptx]

## Slide 1
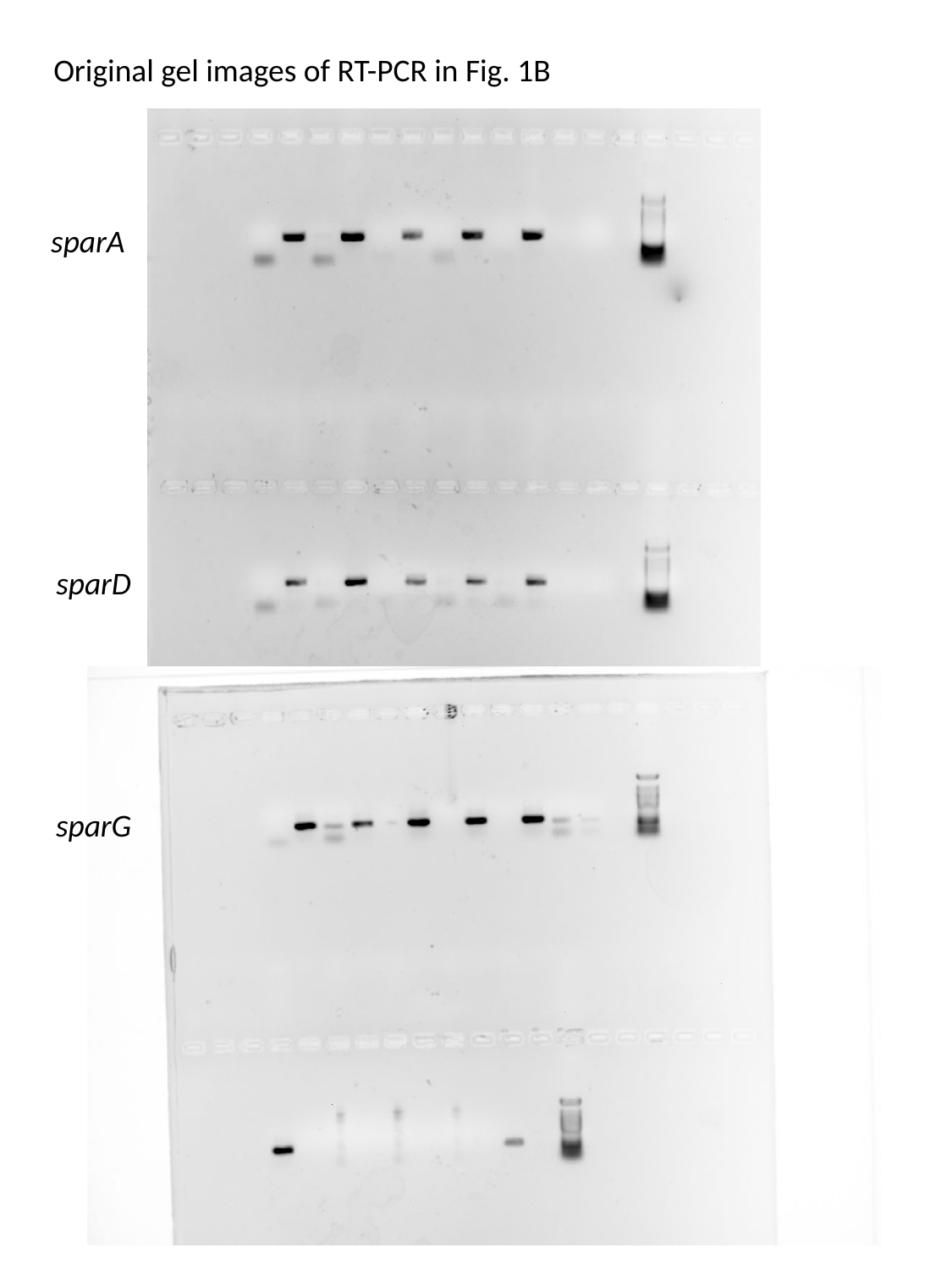

Original gel images of RT-PCR in Fig. 1B
sparA
sparD
sparG

## Slide 2
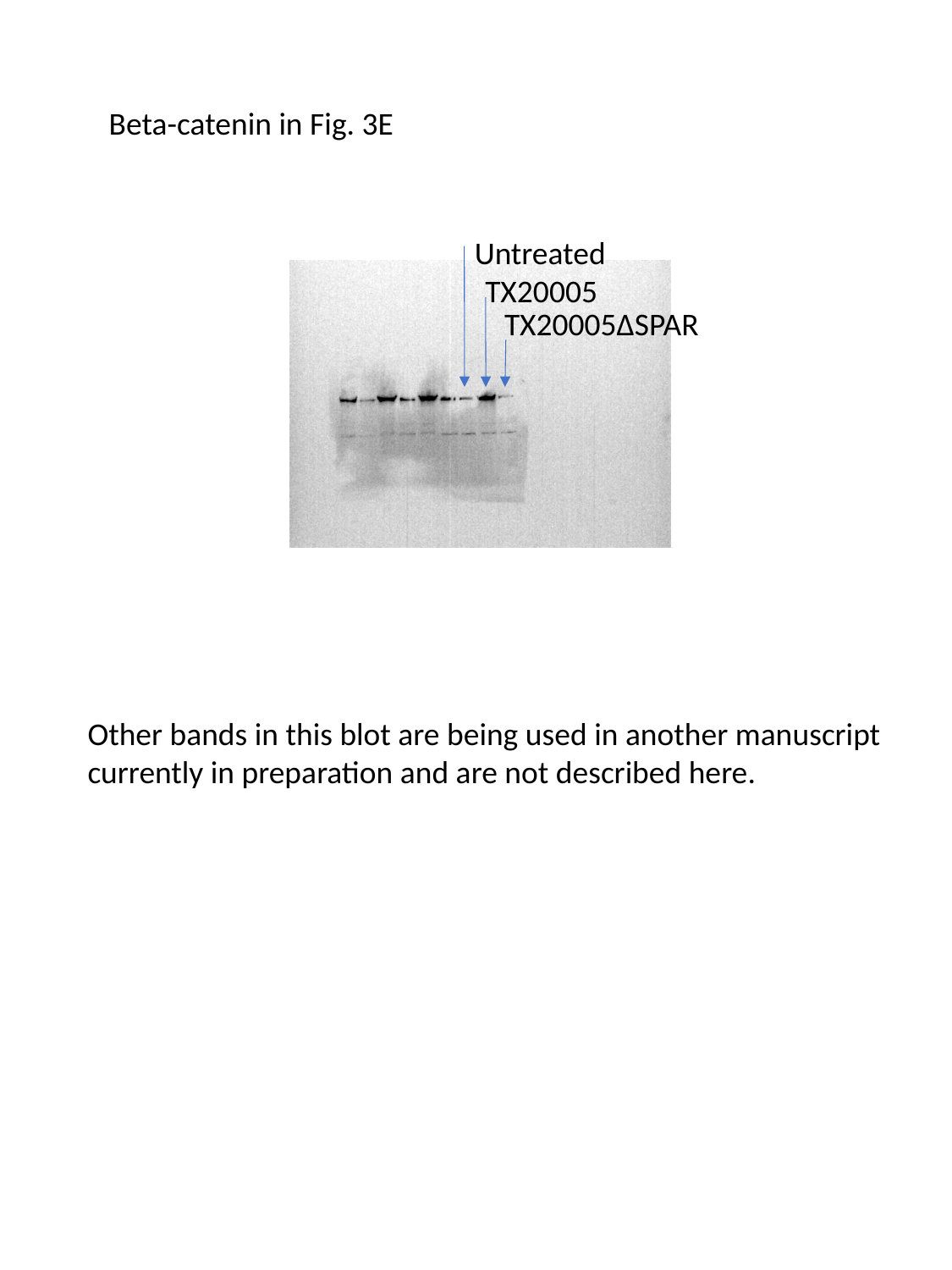

Beta-catenin in Fig. 3E
Untreated
TX20005
TX20005∆SPAR
Other bands in this blot are being used in another manuscript currently in preparation and are not described here.

## Slide 3
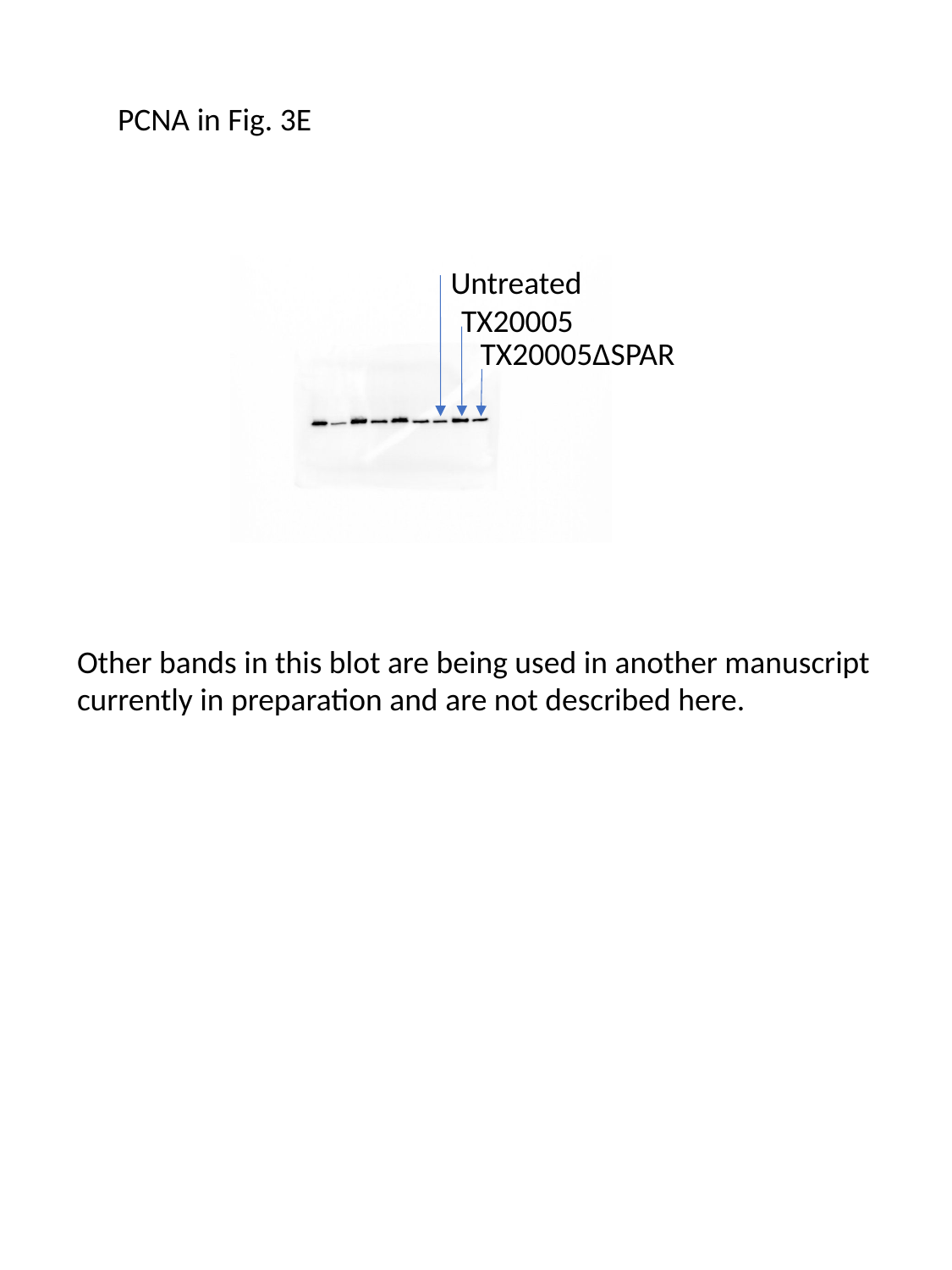

PCNA in Fig. 3E
Untreated
TX20005
TX20005∆SPAR
Other bands in this blot are being used in another manuscript currently in preparation and are not described here.

## Slide 4
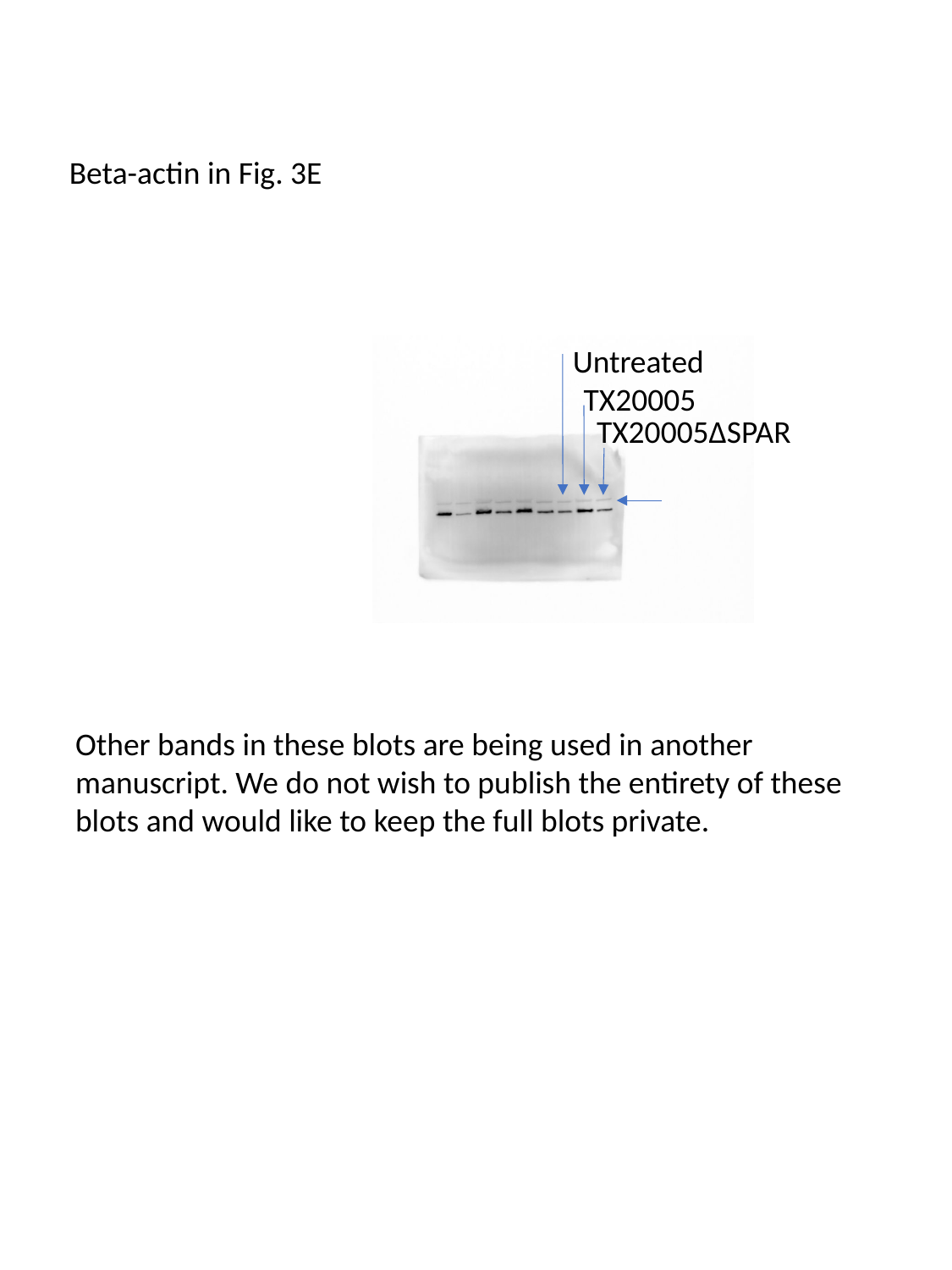

Beta-actin in Fig. 3E
Untreated
TX20005
TX20005∆SPAR
Other bands in these blots are being used in another manuscript. We do not wish to publish the entirety of these blots and would like to keep the full blots private.

## Slide 5
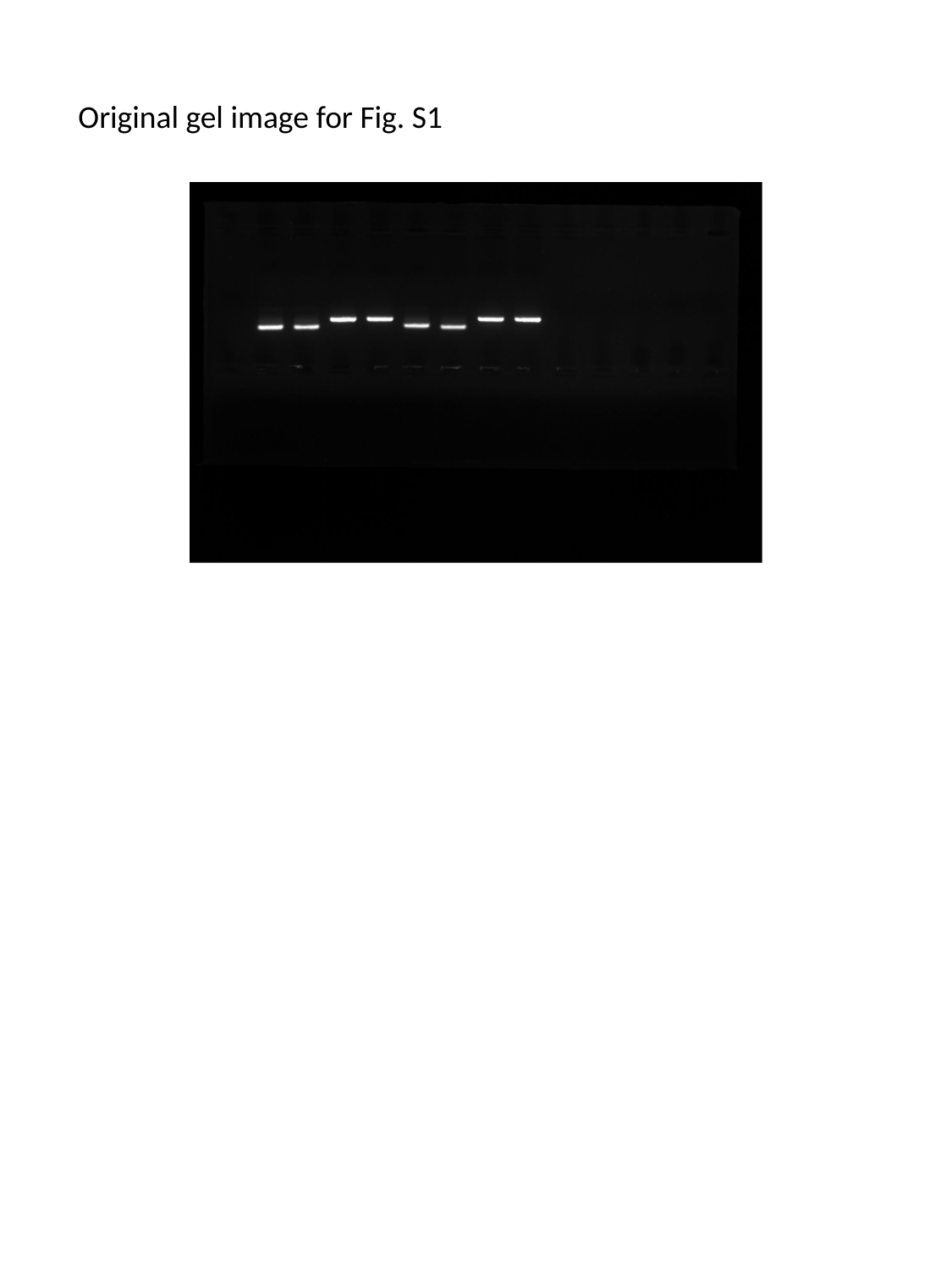

Original gel image for Fig. S1

## Slide 6
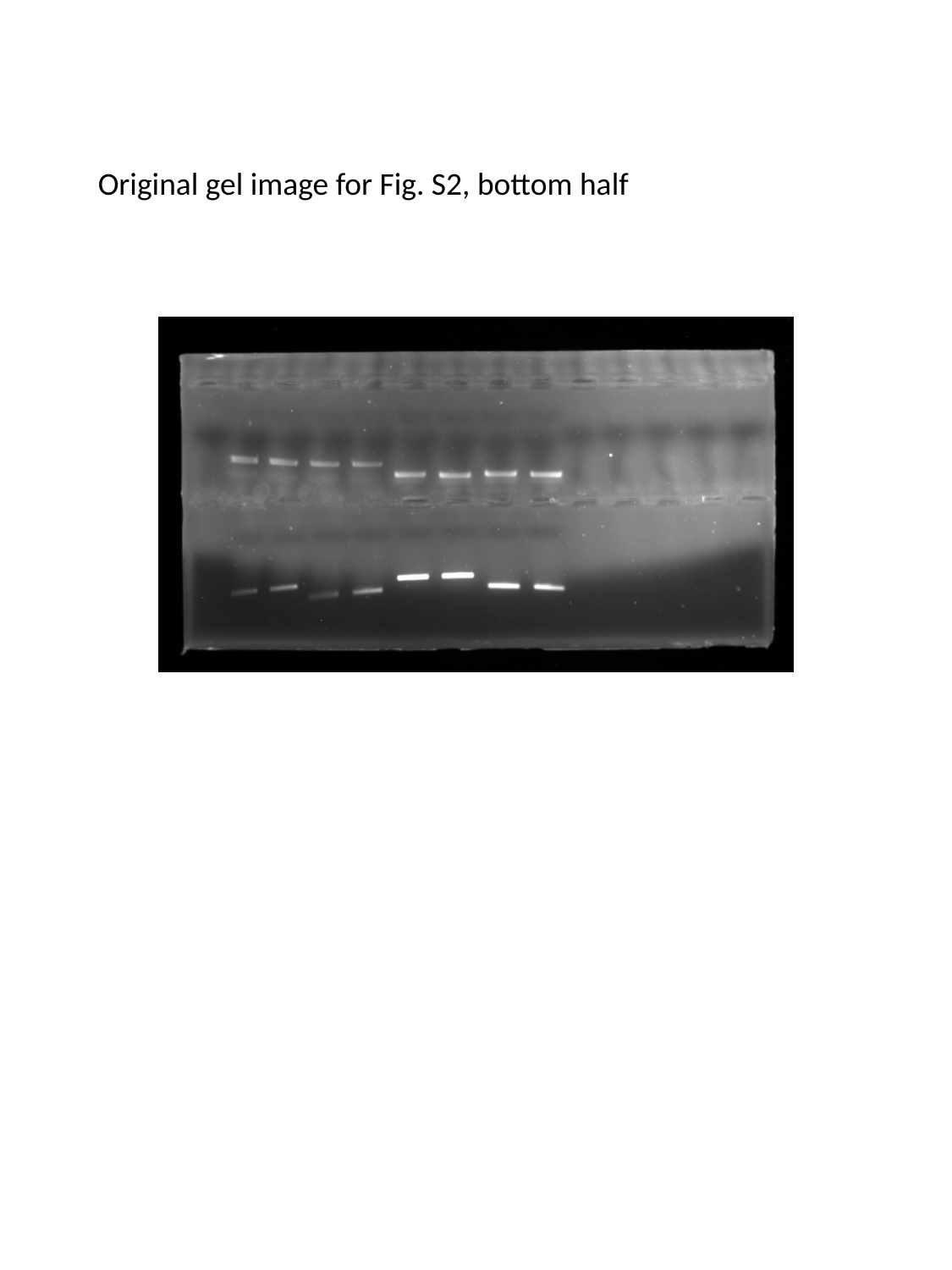

Original gel image for Fig. S2, bottom half

## Slide 7
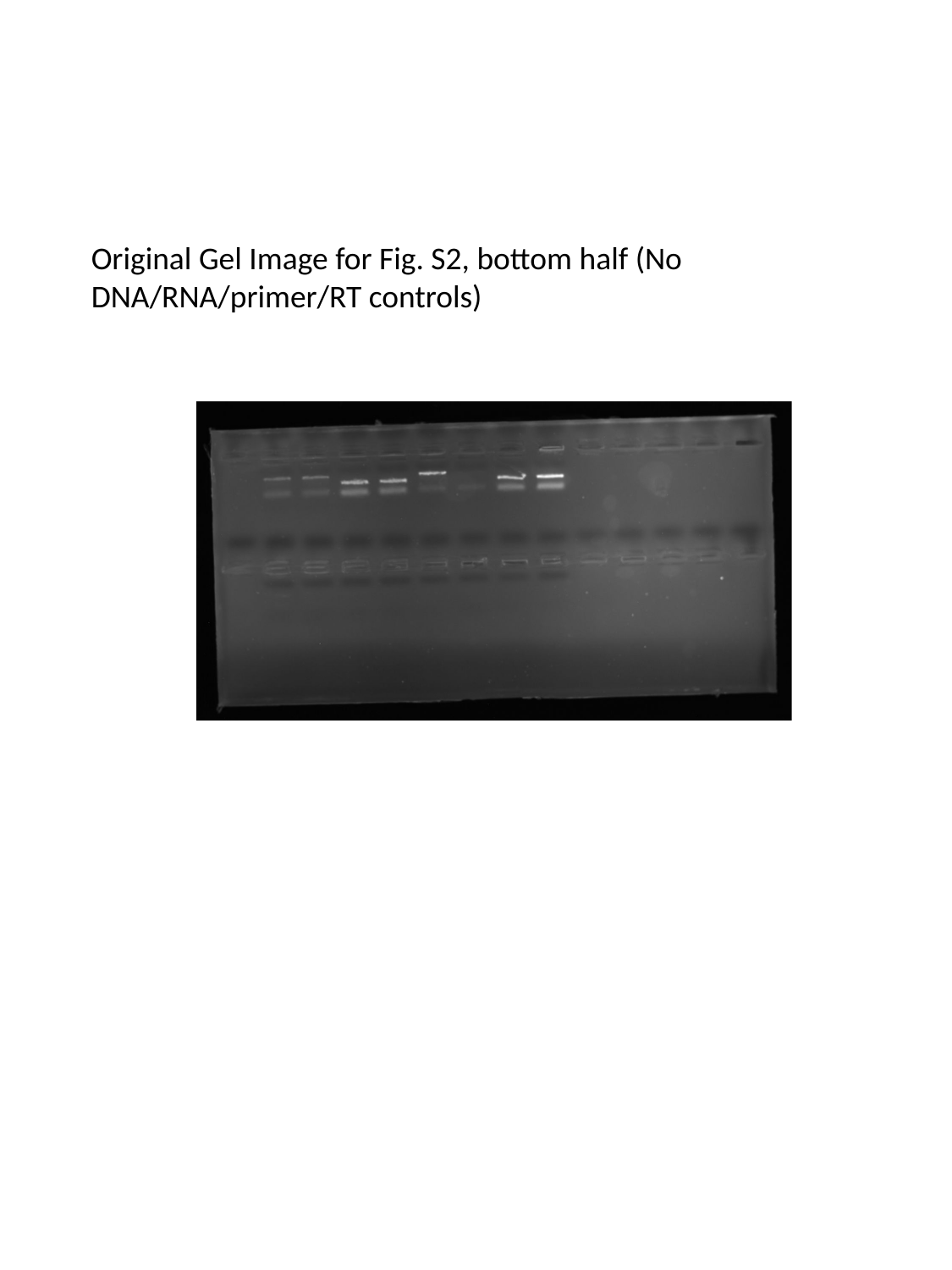

Original Gel Image for Fig. S2, bottom half (No DNA/RNA/primer/RT controls)

## Slide 8
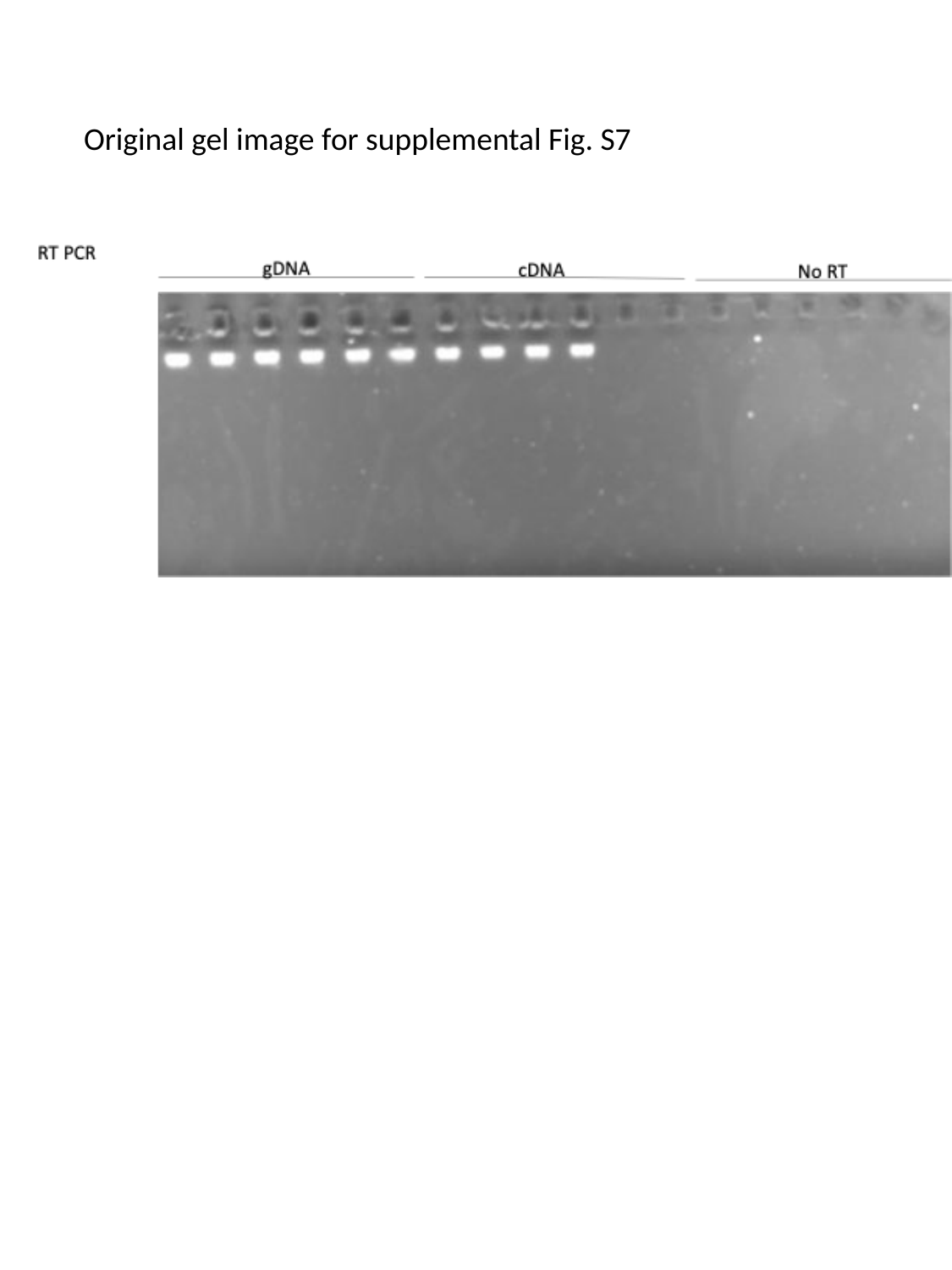

Original gel image for supplemental Fig. S7
